# A Transgenic 5xFAD-M Line of Mice for Dendritic Spine Morphology Analysis in Alzheimer’s Disease

**DOI:** 10.3390/brainsci13020307

**Published:** 2023-02-10

**Authors:** Anastasiia Ilina, Natalia Linkova

**Affiliations:** 1 Laboratory of Molecular Neurodegeneration, Peter the Great Saint Petersburg State Polytechnic University, 195251 Saint Petersburg, Russia; 2 Department of Biogerontology, Saint Petersburg Institute of Bioregulation and Gerontology, 197110 Saint Petersburg, Russia; 3 The Laboratory “Problems of Aging”, Belgorod National Research University, 308015 Belgorod, Russia

**Keywords:** Alzheimer’s disease, 5xFAD-M, GFP, spine morphology, confocal microscopy, neuron visualization

## Abstract

Cognitive impairments are closely related to synaptic loss in Alzheimer’s disease (AD). Functional changes in synaptic contacts are reflected in dendritic spine morphology. Visualization of neurons for morphological studies in vivo is complicated by the fixed brain slice staining or expensive adeno-associated virus injections. We created a transgenic 5xFAD-M line of mice with AD-associated mutations and expressed GFP protein in single neurons of the brain. This mouse model of AD is a useful tool for the simplified visualization of the hippocampal neurons’ morphology in vivo without additional staining manipulations. The progressive elimination of mushroom spines was demonstrated in 5xFAD-M mice between 4 and 5 months of age. Five-month-old 5xFAD-M male and female mice showed change both in the total density and the mushroom spines number compared to sex-matched control. We conclude 5xFAD-M mice can be a useful AD model for studying the mechanisms of synaptic pathology under neurodegenerative conditions and evaluating the effects of potential therapeutic agents on spine morphology as crucial aspect of memory loss in AD.

## 1. Introduction

Alzheimer’s disease (AD) is a progressive neurodegenerative disorder that is the most common cause of dementia [1]. Clinicopathological studies suggest that initial degenerative impairments develop 10–15 years before the onset of clinical symptoms [2]. Neurodegenerative processes are known to be developed in the entorhinal cortex, hippocampus, temporal lobe, parietal cortex, and prefrontal cortex in initial stages of AD leading to memory impairments and cognitive decline [3,4]. Neurodegenerative processes’ progression is characterized by increased involvement of the entire neocortex with some subcortical regions involved, including the striatum, thalamus, hypothalamus, and cerebellum as well as the reticular formation of the medulla, enhancing cognitive decline and leading to behavioral and speech deficits, visual and spatial disorientation, and impaired motor activity [4,5]. Depressive symptoms, mood changes, psychosis, anxiety, hallucinations, aggression, and circadian rhythm disruption are reported in the patients with disease progression, leading to complete dependence on others in daily activities and comprehensive nursing care [2]. AD is associated with the progressive disability of patients, causing death 5–12 years after symptom onset [6].

According to the amyloid hypothesis, the most prevalent theory of AD development, the misfolding of the amyloid β protein (Aβ), causes neurodegeneration and Aβ accumulation in the form of extracellular amyloid plaques in the brain is believed to be the main histopathological characteristic of Alzheimer’s disease [7,8]. The Aβ protein is formed during the sequential cleavage of the transmembrane amyloid precursor protein (APP) [9]. Eight isoforms of APP are formed during alternative splicing. An Aβ form with 695 amino acid residues is expressed in the brain [10]. Toxic Aβ forms are the result of APP proteolysis by β- and γ-secretases. Presenilin proteins (PSEN) of the 1st and 2nd types perform enzymatic function in the γ-secretase multi-protein complex [11]. Autosomal dominant mutations in the APP, PSEN1, and PSEN2 genes promote amyloidogenic cleavage of APP with β- and γ-secretases. These mutations associated with the familial Alzheimer’s disease (FAD) development [12].

It was shown that Aβ accumulation was poorly correlated with cognitive impairments in AD [13]. In contrast, cognitive dysfunction in mice is closely correlated with a decrease in the number of synaptic contacts in the hippocampal neurons [14,15].

The postsynaptic elements of synapses called dendritic spines. Dendritic spine morphology depends on their functional activity. In particular, the spine head participates in synaptic communication that associates with molecular processes of new proteins’ synthesis in postsynaptic density (PSD) [16]. Increasing functional spine activity associates with increasing protein synthesis and PSD growth that provide spine head growth. Spine morphology tightly correlates with the neuroplasticity in the brain, which underlies the processes of learning and memory [17]. Furthermore, changes in dendritic spines occur in the early AD stages, prior to neuronal death and amyloid plaque formation [18].

Although the Alzforum database contains 197 mouse models of AD, the closest correlation of cognitive impairments with the synaptic loss is observed in the transgenic line of 5xFAD mice (B6SJL-Tg (APPSwFlLon, PSEN1 * M146L * L286V)6799Vas/Mmjax, Stock No: 34840-JAX) [19]. This line contains 5 mutations associated with familial AD. The genome of 5xFAD mice contains transgenic inserts. The human APP gene contains three missense mutations Swedish K670N/M671L, Florida I716V, and London V717I, which are grouped near the sites of APP cleavage and contribute an increase of the Aβ production [20]. 5xFAD mice demonstrate memory impairments in Y-maze test [20], Morris water maze test [21], novel object recognition test [22], and fear conditioning test [23].

Despite the abundance of various AD mouse models, the visualization of neurons for morphological studies is complicated by the necessity of pre-fixed brain staining or adeno-associated viruses inject in vivo [24]. The GFP-M transgenic line of mice (Tg (Thy1-EGFP) MJrs/J, C57BL/6J background, Stock No: 007788) is characterized by selective expression of the green fluorescence protein (GFP) under the control of the neuron-specific promoter from the *thy1* gene in a single neuron of the brain, including the hippocampal CA1 pyramidal neurons [25]. GFP expression is not detectably toxic and has no discernible effect on synaptic structure, which offers great opportunities for simplified morphological studies of synapses without brain slices staining or AAV injections in vivo [25]. We have cross-bred 5xFAD mice with M mice in the present study, which made it possible to identify the hippocampal neurons with minimal technical intervention and to analyze morphological changes in dendritic spines of hippocampal CA1 pyramidal neurons affected by neurodegeneration in AD.

## 2. Materials and Methods

***Animals.*** 5xFAD and M mice obtained from the Jackson Laboratory (Bar Harbor, ME, USA) were used to create a transgenic 5xFAD-M line of mice. Four to five mice per cage were kept under a 12-h light/dark cycle in a vivarium of Laboratory of Molecular Neurodegeneration, Peter the Great Saint Petersburg State Polytechnic University. Food and water were provided ad libitum.

***Experimental design.*** The animals were combined into the following experimental groups to analyze the dendritic spine morphology in 4 and 5-month old male 5xFAD-M mice: 1—M mice (*n* = 5; 4 month); 2—5xFAD-M mice (*n* = 5; 4 month); 3—M mice (*n* = 5; 5 month); 4—5xFAD-M mice (*n* = 5; 5 month). The animals were combined into the following experimental groups to analyze the dendritic spines morphology in male and female 5xFAD-M mice: 1—M mice (*n* = 5; male); 2—5xFAD-M mice (*n* = 5; male); 3—M mice (*n* = 5; female); 4—5xFAD-M mice (*n* = 5; female). This age of mice was chosen based on the literature data about the AD progress in 5xFAD mice [20].

***Genotyping.*** Mice genotype was determined by standard genotyping procedure with genomic DNA isolating from the tail tip. APP, PSEN1, and GFP transgenes were determined by PCR with the following priming pairs: APP (forward—AGG ACT GAC CAC TCG ACC AG, reverse—CGG GGG TCT AGT TCT GCA T), PSEN (forward—AATAGAGAACGGCAGGAGCA, reverse—GCCATGAGGGCACTAATCAT), GFP (forward—ACA GAC ACA CAC CCA GGA CA, reverse—CGG TGG TGC AGA TGA ACT T), internal positive control (forward—CTA GGC CAC AGA ATT GAA AGA TCT, reverse—GTA GGT GGA AAT TCT AGC ATC ATC C).

***Tissue preparation.*** All animals were anesthetized by intraperitoneal 250 mg/mL urethane (Sigma-Aldrich, St. Louis, MO, USA) injection and were perfused transcardially with 10–20 mL PBS and 30–50 mL 4% paraformaldehyde (PFA) (3 mL/min) after verifying the sufficient depth of anesthesia. The brains of decapitated mice were rapidly removed, post-fixated in a 4% PFA for during 1 week at 4 °C, and were cut into 40 μm brain slices in 1xPBS by means of Lancer Vibratome Series 1000 Sectioning System 054,018 (USA). These slices were mounted in Aqua Poly/Mount (Polysciences, Inc., Philadelphia, PA, USA, Cat# 18606).

***Imaging.*** All confocal images were captured using laser scanning confocal microscope ThorLabs with 100× objective lens (UPlanSApo; Olympus, Tokyo, Japan). Images were collected at a resolution of 1024 × 1024 pixels, with 0.067 μm/pixel. Z stacks were assembled by 4–8 μm images (Z interval 0.2 μm). GFP expression was quantified in ImageJ software by measuring the integrated density (ID) in relation to area of interest. The microphotographs were processed by the Richardson–Luci algorithm in DeconvolutionLab plugin to analyze the dendritic spine morphology. The point spread function was established in the Huygens program.

***Dendritic spine morphology analysis.*** Dendritic spine morphology was analyzed in 10–15 fragments of CA1 secondary apical dendrites of each mice using NeuronStudio software package [26]. The following cutoff values were used for dendritic spine classification: thin ratio = 2.5, HNRcrit = 1.3, and HDcrit = 0.35 μm. DSD was calculated as ratio of total spines number to 10 μm of dendritic length, and also mushroom (MS), thin (TS), and stubby (SS) spines number was calculated relative to the total spines number and was used for characterization of spine morphology.

***Statistical analysis.*** The data were assessed by Shapiro–Wilk test, two-way ANOVA test with post-hoc Tukey HSD, and Mann–Whitney U test. Pre-analytical data processing was carried out by MS Excel. Statistical analysis was performed by Statistica 12 and GraphPad Prism 8 software. The results were presented as mean ± SEM. The significance level was *p* < 0.05 (*), *p* < 0.01 (**), *p* < 0.001 (***); ns—non significant.

## 3. Results

### 3.1. Creating of a Transgenic Line of 5xFAD-M Mice

We cross-bred 5xFAD mice with M mice that have expressed eGFP protein in single neurons of the brain, including the pyramidal neurons of the hippocampus, to analyze dendritic spine morphology of hippocampus in AD. GFP was expressed in pyramidal neurons of 5xFAD-M mice under control of neuron-specific Thy1 promotor. The Thy1 promotor-driven GFP expression did not affect synaptic structures, was not toxic [25], and provided visualization of neuron morphology in fixed brain slices without staining or injecting adeno-associated viruses.

**Figure 1:**
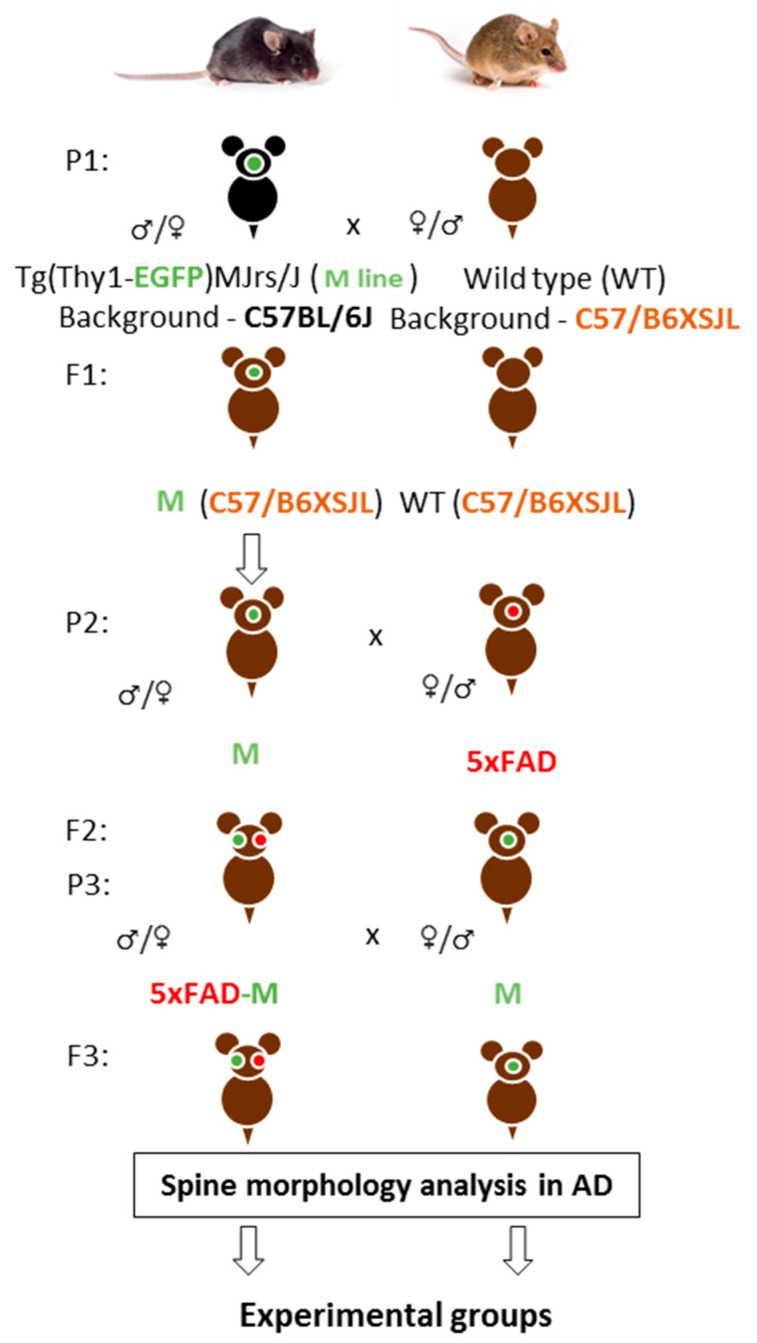
A scheme of cross-breeding 5xFAD and M mice to create a 5xFAD-M transgenic line of mice. P—parents, F—hybrids.

Firstly, we crossed M and wild-type (WT) mice on the C57/B6XSJL background. Then, we had animals with three APP, PSEN1, GFP transgenes (5xFAD-M) on C57/B6XSJL background and animals with only the GFP transgene (M) on C57/B6XSJL and C57BL/6J backgrounds in the first filial generation after M mice and 5xFAD mice-crossing on C57/B6XSJL background. Second filial generation was taken by the 5xFAD-M and M mice-crossing on C57/B6XSJL background. The 5xFAD-M and M mice of the third and fourth generations were used for dendritic spine morphology analysis (Figure 1).

**Figure 2:**
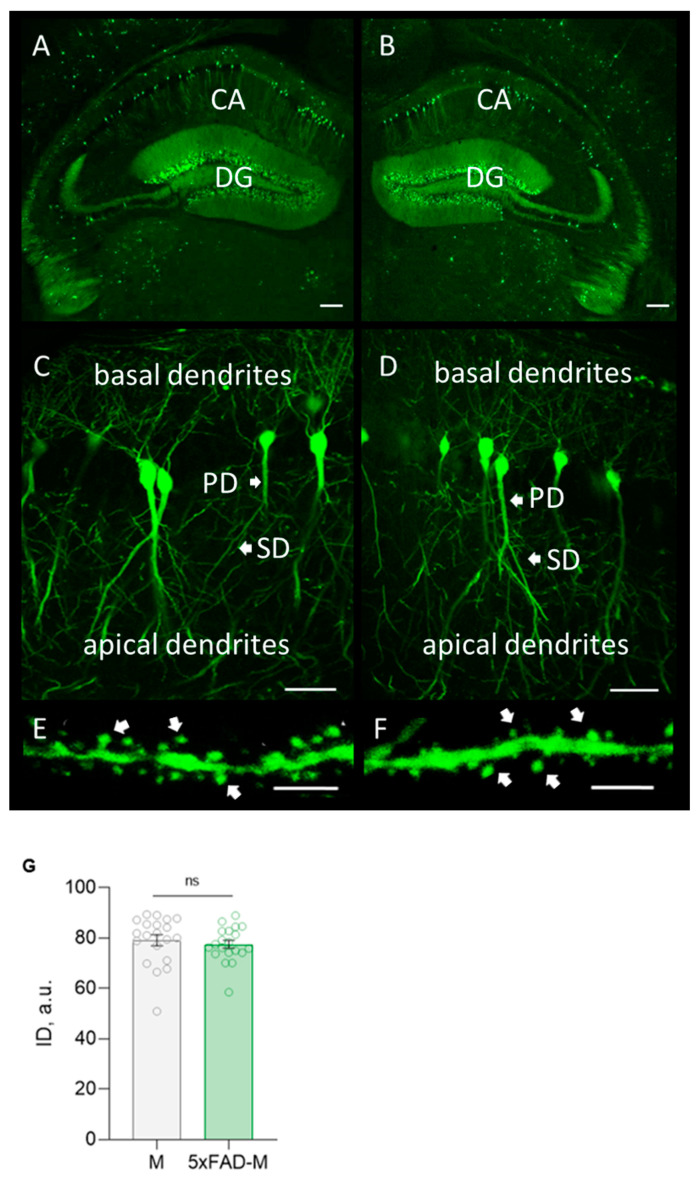
Confocal microscopy of GFP expression in hippocampal slices from a 5xFAD-M and M mouse brain. (**A**,**B**) Hippocampal and dentate gyrus (DG) section in M (**left**) and 5xFAD-M (**right**) mouse brain. Scale bar 200 μm. (**C**,**D**) Basal and apical dendrites of pyramidal neuron in CA1 region of the hippocampus of M (**left**) and 5xFAD-M (**right**) mice. Scale bar 50 μm. (**E**,**F**) Spines of secondary apical dendrite with dendritic spines of hippocampal pyramidal neuron in CA1 region of M (**left**) and 5xFAD-M (**right**) mouse. Scale bar 10 μm. (**G**) Integrated density of GFP expression.

The highly GFP expression in neurons of a hippocampal formation from a transgenic 5xFAD-M line of mice was revealed by morphological analysis (Figure 2). It was no difference in the integrated density of GFP expression in 5xFAD-M and M mice (Unpaired *t*-test: t = 0.5942, df = 38, *p* = 0.5559).

Thus, we create a transgenic 5xFAD-M line of mice for simplify the visualization of the hippocampal neurons’ morphology without additional staining manipulations.

### 3.2. 5xFAD-M Mice Demonstrate Elimination of Mushroom Spines between 4 and 5 Months of Age

**Figure 3:**
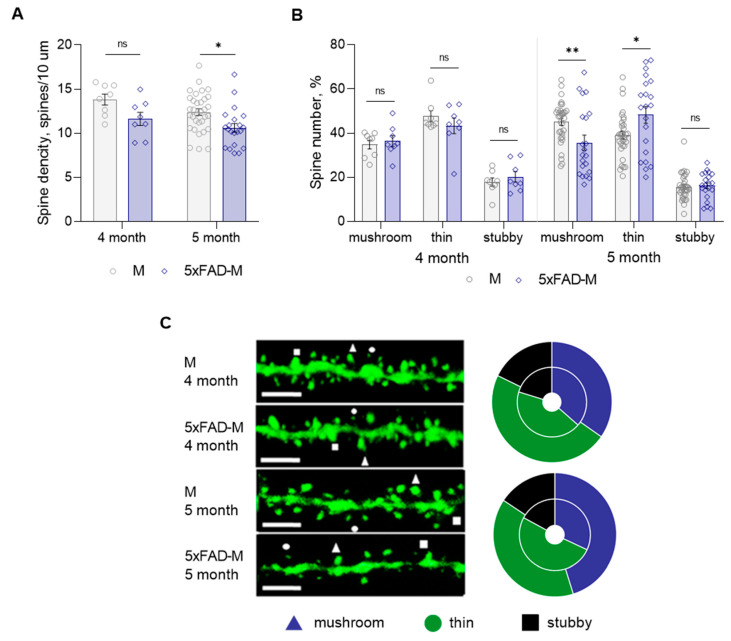
Changes in dendritic spine morphology in 4- and 5-month-old mice of new transgenic 5xFAD-M line compared to age-matched M mice (*n* = 5 in each group). (**A**) Spine density of CA1 secondary dendrites in hippocampus of mice; (**B**) Relative spines number of CA1 secondary dendrites in hippocampus of mice. (**C**) **Left**—Confocal images of the CA1 secondary dendrites in hippocampus of mice. Confocal microscopy. Scale bar, 10 μm. **Right**—Distribution diagrams of spine types in hippocampus of 5xFAD-M (inside) and M (outside) mice. *, **—*p* < 0.05, *p* < 0.01 compared to M mice.

Total spine density and dendritic spine morphology in CA1 region of 4- and 5-month-old 5xFAD-M mice hippocampus were assessed with comparison to old M mice. We did not find statistically significant differences between DSD in 4-month-old 5xFAD-M and M mice (two-way ANOVA: F (1,66) = 10.31, *p* = 0.002; Tukey’s test: q = 2.865, DF = 66, *p* = 0.189), but we observed a trend to its decline (Table 1; Figure 3A,C). Differences between a number of mushroom (Mann–Whitney U test: U (67, 69) = 31, *p* = 0.959), thin (Mann–Whitney U test: U (72.50, 3.50) = 27.5, *p* = 0.665), and stubby (Mann–Whitney U test: U (65, 71) = 29, *p* = 0.798) spines of CA1 neurons in 4-month-old 5xFAD-M and M mice were likewise not observed (Table 1; Figure 3B,C). DSD in 5-month-old 5xFAD-M mice was reduced by 15% (two-way ANOVA: F (1,66) = 10.31, *p* = 0.002; Tukey’s test: q = 2.865, DF = 66, *p* = 0.022) compared to the control M mice (Table 1; Figure 3A,C). The spine density decreased due to the loss of mushroom spines by 21% (Mann–Whitney U test: U (1056, 429) = 198, *p* = 0.008) in 5-month-old 5xFAD-M mice compared to the M mice. The number of thin spines increased by 19% (Mann–Whitney U test: U (794, 691) = 233, *p* = 0.044) in 5-month-old 5xFAD-M mice compared to the control M mice. The number of stubby spines did not differ (Mann–Whitney U test: U (860, 625) = 299, *p* = 0.405) in 5-month-old 5xFAD-M and M mice (Table 1; Figure 3B,C).

Thus, we have demonstrated the progressive elimination of mushroom spines occurred between 4 and 5 months of age in 5xFAD-M mice.

### 3.3. Dendritic Spine Pathology in Male and Female 5xFAD-M Mice

**Figure 4:**
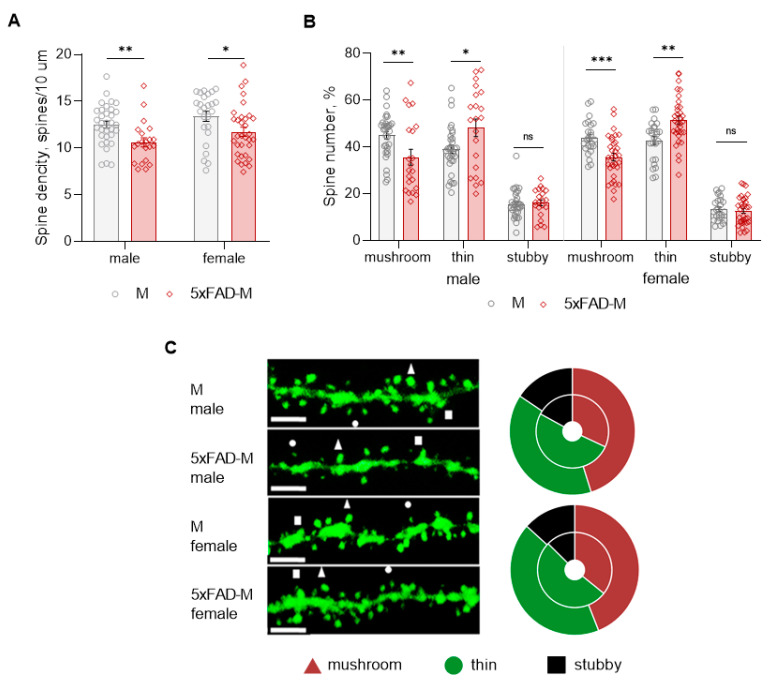
Changes in dendritic spine morphology in 5xFAD-M and M male and female mice (*n* = 5 in each group). (**A**) Spine density of CA1 secondary dendrites in hippocampus of mice; (**B**) Relative spines number of CA1 secondary dendrites in hippocampus of mice; (**C**) **Left**—Confocal images of the CA1 secondary dendrites in mice. Confocal microscopy. Scale bar, 10 μm. Right—Distribution diagrams of spine types in hippocampus of 5xFAD-M (inside) and M (outside) mice. *, **, ***—*p* < 0.05, *p* < 0.01, *p* < 0.001 compared to M mice.

Dendritic spine density of CA1 neurons in 5xFAD-M males was reduced by 15% (Mann–Whitney U test: U (1082, 403) = 172, *p* = 0.002) compared to M mice (Table 2; Figure 4A,C). Dendritic spine density was reduced due to the elimination of mushroom spines by 21% (Mann–Whitney U test: U (1056, 429) = 198, *p* = 0.008) in 5xFAD-M males (Table 2; Figure 4B,C). The number of thin spines increased by 19% (Mann–Whitney U test: U (794, 691) = 233, *p* = 0.044) in 5xFAD-M males compared to the control M males. There was no difference (Mann–Whitney U test: U (860, 625) = 198, *p* = 0.405) in the stubby spine number in 5xFAD-M and M males (Table 2; Figure 3B,C).

Dendritic spine density of CA1 neurons in 5xFAD-M females decreased by 12% (Mann–Whitney U test: U (894, 817) = 256, *p* = 0.014) compared to the control mice (Table 2; Figure 4A,C). Dendritic spine density of neurons in 5xFAD-M females was reduced due to the elimination of mushroom spines by 19% (Mann–Whitney U test: U (950, 761) = 200, *p* = 0.0006) (Table 2; Figure 4A,C). The thin spine number increased by 17% (Mann–Whitney U test: U (536.5, 1175) = 211.5, *p* = 0.001) in 5xFAD-M females compared to the control M females. There was no difference in the stubby spine number in 5xFAD-M and M females (Mann–Whitney U test: U (778, 933) = 372, *p* = 0.530) (Table 2; Figure 3B,C).

Thus, 5-month-old 5xFAD-M male and female mice showed differences both in the total density and the number of mushroom spines compared to the sex-matched control mice.

## 4. Discussion

Cognitive dysfunction is closely related to a decrease of the number of synaptic contacts in hippocampal neurons in AD. Spine loss is an early and important pathological event that occurs long before neuronal death in AD. Dendritic spine morphology reflects pathomorphological changes in a synapse during this disease. The visualization of dendritic spine morphology in 5xFAD mouse model of AD is not possible to carry out without additional techniques such as staining or adeno-associative virus injections. The solution to this problem can be created by using GFP-expressing transgenic AD mice. It is possible to generate transgenic mice expressing multiple spectral variants of GFP in different pathologies as described in the literature [18,27,28,29].

The transgenic line of YFP (J16)-R6/2 mice has been made for directly evaluating early axonal abnormalities in vivo in Huntington’s disease (HD). Authors demonstrated a dying-back pattern of degeneration for cortical projection neurons affected in HD, suggesting that axons represent an early and potentially critical target for mutant Huntington toxicity [27]. In another study, thy1-GFP M mice were crossed with presenilin-1-M146V knock-in (PS1-KI) mice (AD model) to generate PS1-KI-GFP mice for in vivo hippocampal mushroom spine analysis. By creating new PS1-KI-GFP transgenic mice, authors were successful in revealing the normalized action of pridopidine to mushroom spine stability in vivo [28]. Using of this transgenic line of mice jointly with another GFP-expressing transgenic APP-KI-GFP mouse model of AD allowed them evaluate the ability of the novel nSOC-positive modulator (NSN21778) to save mushroom spine loss in hippocampal slices [29]. Finally, a significant loss of spines on basal dendrites in the somatosensory and prefrontal cortices was found in 6-month-old 5XY female by cross-breeding 5xFAD mice with YFP-H line of mice [30]. 

We cross-bred 5xFAD mice with GFP mice to analyze dendritic spine morphology of the hippocampus in a 5xFAD mouse model of AD. Interestingly, the 5xFAD/Thy1-GFP-M line of mice was successfully used by Jianping Zhang and colleges for axonopathy assessment in an early stage of 5xFAD/GFP mice. Authors showed that among GFP-labeled axons, GFP-labeled axonopathy underwent early alterations in the lateral septal nucleus, subiculum, and mammillary nucleus in the 5xFAD/GFP mouse model. It is important to point out that authors have not been evaluating the dendritic spine morphology in this mouse model [31].

We have observed in the present study the decrease in dendritic spine density at 4 and 5 months of age in 5xFAD-M mice, thus confirming the current data on synaptic dysfunction in 5xFAD mice [20] and morphological changes in neurons [14,32]. Moreover, decreasing the number of mushroom spines and increasing of the number of thin spines have been performed on the hippocampal CA1 neurons of 5xFAD-M line of mice, suggesting that the maturation and elimination of spines constitute the dynamic process of spine shape conversion depending on functional state [33]. Mushroom spines with a large head and small necks have the longest life span, and form strong synaptic contacts, and therefore are considered to be the most functionally active protrusions and the sites of long-term memory storage [15,34]. Thin spines with a smaller head are more dynamic and thought to be the ‘’learning spines’’ involved with new memory formation [35,36,37]. Synaptic plasticity enhancing is accompanied by growing the spine head and the process of thin to mushroom spines conversion, due to the synthesis of new protein molecules on post-synaptic terminals [38]. Dendritic spine shrinkage and elimination was shown in the cortex and hippocampus of AD patients long before cognitive impairment [15,34,35,39,40]. Furthermore, spine loss and progressive changes of mushroom spines in CA1 hippocampal neurons were observed in ex vivo hippocampal slices prepared from 7- or 14-day-old mice that were kept in culture for 15 or 20 days [41]. It is therefore likely that the findings indicate the development of the processes of mushroom into thin dendritic spines conversion in hippocampal CA1 neurons of 5xFAD-M mice, reflecting the dendritic spine shrinkage with decreased synaptic plasticity revealed in 5xFAD mice [42,43,44].

We have shown the elimination of the most functional dendritic spines of mushroom type in the male and female of 5xFAD-M mice compared to sex-matched mice of the control M line. According to Holly Oakley’s data, young 5xFAD females from 2 to 5 months of age tended to have slightly higher Aβ42 levels than age-matched males, but this trend appeared to diminish at older ages. Furthermore, significant sex-dependent differences in the levels of presynaptic markers synaptophysin have not been observed in whole-brain homogenates from 5xFAD mice aged from 2 to 12 months old [20].

Thus, 5xFAD-M line of mice can be a useful tool for studying the mechanisms of the synaptic pathology that underlies memory loss in AD. It should be pointed out that the level of technological development in the field of neurobiology allows carrying out experiments in real time. For instance, Lin X and colleagues have used head-mounted, miniature fluorescent microscopes (“miniscope”) on freely moving animals to examine the hippocampal neural ensemble activities during behavioral tasks in a triple-transgenic mouse model of AD. Importantly, authors have stereotaxically injected AAV1-CamKII (0.4)-GCaMP6f in order to image in vivo calcium transients of hippocampal CA1 excitatory neurons [45]. Taking the spines as dynamic structures into account [46], real-time changes in spine morphology attract scientific. In this case, using the 5xFAD-M line of mice will eliminate the need to use AAV constructs to visualize neurons, an important advantage in the study of the functional activity of neural networks in the freely moving animal amid the development of AD-associated neurodegenerative processes.

Due to the fact that an effective drug for the treatment of AD has not yet been found, the investigation into the effect of potentially therapeutic agents on the development of the synaptic pathology in AD is an important aspect of the development of the new effective compounds for disease correction. Thus, a transgenic 5xFAD-M line of mice is a useful tool for studying the synaptic pathology under neurodegeneration conditions, and can be used for evaluation to the neuroprotective effect of potentially therapeutic compounds for AD treatment as described [47].

## 5. Conclusions

We have cross-bred 5xFAD mice with M mice for analyzing morphological changes in dendritic spines of hippocampal CA1 pyramidal neurons. 5xFAD-M mice demonstrate a decrease in dendritic spine density, progressive elimination of mushroom spines, and an increase in the number of thin spines between 4 and 5 months of age. Five-month-old 5xFAD-M male and female mice showed changes in both the total density, mushroom, and thin spines number compared to sex-matched control. We conclude 5xFAD-M mice can be a useful AD model for studying the mechanisms of synaptic pathology under neurodegenerative conditions and evaluating the effects of potential therapeutic agents on spine morphology affected by neurodegeneration in AD.

## 6. Limitations and Future Directions

It was necessary to use brain slice thicknesses of no more than 40 μm for qualitative visualization of the dendritic spine morphology on fixed slices of mice brain.

It was necessary to carry out qualitative perfusion of mice for blood to PBS substitution preceding PFA fixation. Using the heparinized PBS [48] and buffered picric acid-paraformaldehide [49] makes it possible to improve the quality of perfusion and fixation of mouse brains.

The 5xFAD-M line of mice will be used for future study of the dendritic spine morphology and activity of neuronal networks in behavioral tests in vivo.

## Figures and Tables

**Table 1:** Parameters of dendritic spine morphology in 4- and 5-month-old 5xFAD-M mice.

Age	Indicators	M Mice	5xFAD-M Mice
4-month	DSD	13.82 ± 0.62	11.64 ± 0.74
	MS	34.83 ± 2.02	36.44 ± 2.48
	TS	47.68 ± 2.48	43.29 ± 3.51
	SS	17.86 ± 1.88	20.26 ± 2.35
5-month	DSD	12.40 ± 0.38	10.62 ± 0.49 *
	MS	45.06 ± 1.64	35.64 ± 3.41 **
	TS	39.07 ± 1.78	48.21 ± 3.78 *
	SS	15.61 ± 1.02	16.15 ± 1.30

DSD—dendritic spine density (spines/10 μm), MS—number of mushroom spines (%), TS—number of thin spines (%), SS—number of stubby spines (%); *, **—*p* < 0.05, *p* < 0.01 compared to M mice.

**Table 2:** Indicators of dendritic spine morphology in male and female 5xFAD-M mice.

Sex	Indicators	M Mice	5xFAD-M Mice
Males	DSD	12.52 ± 0.38	10.62 ± 0.49 **
	MS	45.06 ± 1.64	35.64 ± 3.41 **
	TS	39.07 ± 1.78	48.21 ± 3.78 *
	SS	15.61 ± 1.02	16.15 ± 1.30
Females	DSD	13.39 ± 0.53	11.74 ± 0.49 *
	MS	43.94 ± 1.44	35.60 ± 1.62 ***
	TS	42.65 ± 1.69	51.38 ± 1.76 **
	SS	13.31 ± 0.98	12.62 ± 1.05

DSD—dendritic spine density (spines/10 μm), MS—number of mushroom spines (%), TS—number of thin spines (%), SS—number of stubby spines (%); *, **, ***—*p* < 0.05, *p* < 0.01, *p* < 0.001 compared to M mice.

## Data Availability

The data is unavailable due to privacy.
